# Model for End-Stage Liver Disease (MELD) Score as a Predictor and Monitor of Mortality in Patients with *Vibrio vulnificus* Necrotizing Skin and Soft Tissue Infections

**DOI:** 10.1371/journal.pntd.0003720

**Published:** 2015-04-29

**Authors:** Kuo-Chin Huang, Yao-Hung Tsai, Kuo-Chung Huang, Mel S. Lee

**Affiliations:** 1 Department of Orthopaedic Surgery, Chang Gung Memorial Hospital, Chiayi, Taiwan; 2 College of Medicine, Chang Gung University, Taoyuan, Taiwan; 3 Department of Business Administration (Statistics), Nanhua University, Chiayi, Taiwan; University of Tennessee, UNITED STATES

## Abstract

**Background:**

*Vibrio vulnificus* necrotizing skin and soft tissue infections (VNSSTIs) usually predispose patients with or without preexisting liver disease to septic shock, and then evolve to multiple organ dysfunction syndrome (MODS), thus resulting in high mortality in humans. However, clinicians do not have a valid prediction model to provide a reliable estimate of case-fatality rate when caring for these acutely and/or critically ill patients.

**Methods/Principal Findings:**

We retrospectively analyzed 39 consecutive patients with VNSSTIs (mean age: 65.7 ± 11.3 years) at our institution between 2007 and 2010. All patients were treated with the same protocol. Demographic and clinical characteristics, disease severity on admission, treatment details, and outcomes were collected for each patient and extracted for analyses. We studied the predictive value of the model for end-stage liver disease (MELD), modified MELD including sodium (MELD-Na), and laboratory risk indicator for necrotizing fasciitis (LRINEC) scores for case-fatality. Logistic regression and receiver operating characteristic (ROC) curve analyses were performed. The mean MELD, MELD-Na and LRINEC scores on admission were 15.1 ± 1.1, 17.7 ± 1.1, and 3.4 ± 0.4 points, respectively. After admission, these patients had temporary or progressive deterioration of nearly all their scores and lab values. The area under the ROC curve for the MELD and ΔMELD scoring models were 0.929 (*p* = 0.002) and 0.897 (*p* = 0.005), respectively. An optimal MELD/ΔMELD cutoff value ≥ 20/2 had a good sensitivity and specificity (all > 80%), with a 64/13-fold increased odds for case-fatality. Additionally, the development of severe forms of anemia (*p* = 0.014) and hypoalbuminemia (*p* = 0.019) were associated with an increased case-fatality rate.

**Discussion:**

The MELD/ΔMELD scoring model is an effective risk stratification indicator at the time of admission and also an excellent condition monitor during hospitalization for medical care of acutely and/or critically ill patients with VNSSTIs.

## Author Summary

VNSSTIs have increased significantly over the last two decades, with cases now regularly reported globally. Despite advancing antibiotic and infection control practices, these infections are still highly lethal and disabling diseases. They usually predispose patients to septic shock and then evolve to liver dysfunction and MODS, thus resulting in high mortality in humans. However, clinicians do not have a valid prediction model to provide a reliable estimate of case-fatality rate when caring for these acutely and critically ill patients. MELD scoring system was originally developed to assess the short-term prognosis of cirrhotic patients undergoing the transjugular intrahepatic portosystemic shunt procedure. By using the MEGX test, the strong negative association between the scores and the residual liver function has been confirmed. Therefore, it has been validated and applied to a wide spectrum of clinical scenarios in recent years. In this work, we studied the predictive value of the MELD scoring system for case-fatality in patients suffering VNSSTIs. We have found that the MELD scoring system is an effective risk stratification indicator at the time of admission, and also an excellent condition monitor during hospitalization for medical care of acutely and critically ill patients with VNSSTIs.

## Introduction

Necrotizing skin and soft tissue infections caused by marine bacteria, especially of the genus *Vibrio*, appear to be increasing frequency and recognition [1]. Among these, *Vibrio vulnificus* necrotizing skin and soft tissue infections (VNSSTIs), including primary sepsis with secondary skin manifestation and necrotizing wound infections with/without bacteremia, are highly lethal (case-fatality rate of 50% or more), and require rapid and aggressive treatment [2]. Individuals with preexisting liver disease are 80 times more likely to get sick and more than 200 times more likely to die as a result of contact with *V*. *vulnificus* than a patient with no such disease [3]. The reported case-fatality rate from raw oyster-associated VNSSTIs in Florida among patients with preexisting liver disease was 67% compared with 38% among those who were not known to have liver disease [4]. Besides, the rapidly progressing, fulminant VNSSTIs often lead to septic shock and then evolve to multiple organ dysfunction syndrome (MODS), whose severity accounts for the associated high mortality rate [5]. During sepsis, liver dysfunction is one of the MODS components and usually is associated with a poor prognosis [6]. In fact, the injured liver has been considered one of the main actors in the genesis and amplification of multiple organ failure [6]. Even though liver dysfunction may play a key role in mortality of patients with VNSSTIs, clinicians do not have a valid model to provide a reliable estimate of case-fatality rate for caring these acutely and/or critically ill patients.

Model for end-stage liver disease (MELD) scoring system was originally developed to assess the short-term prognosis of cirrhotic patients undergoing the transjugular intrahepatic portosystemic shunt procedure [7]. Since then, it has been validated and applied to a wide spectrum of clinical scenarios [8]. The MELD scoring system takes into consideration objective parameters such as serum creatinine, the international normalized ratio (INR), and serum bilirubin and is computed with statistically derived coefficients on a continuous scale with no upper or lower limits, thus avoiding many drawbacks of other scoring systems. Using the monoethylglycinexylidide (MEGX) test, Botta et al. [9] had further confirmed that an increase in MELD score is associated with a decrease in residual liver function. Because liver dysfunction is strongly associated with mortality in sepsis [6], the authors hypothesize that the MELD scoring systems and its variants may be good predictors and monitors of case-fatality in patients with VNSSTIs. Therefore, the current study was performed to evaluate the performance of these scoring systems in *V*. *vulnificus*-infected patients and to identify the predictors of case-fatality in this group. The information from this study may be valuable in improving medical care of patients with VNSSTIs, thereby reducing the mortality rate.

## Materials and Methods

### Study subjects and the treatment protocol

This retrospective study included all patients with a diagnosis of VNSSTIs who were treated at our institution between January 2007 and December 2010. *V*. *vulnificus* infection was confirmed by isolating pathogenic bacteria from soft-tissue lesions and/or blood collected immediately after arrival at emergency department. Necrotizing skin and soft tissue infection was defined by either histopathologic or surgical findings such as the presence of necrosis of skin, subcutaneous fat, fascia, or underlying muscles. All patients received the same treatment protocol based on the *in vitro* and *in vivo* experimental evidence reported in the relevant literature [10–12], including specific combination antibiotic therapy, aggressive resuscitation and prompt radical débridement. Using the proposed treatment protocol, the authors had successfully improved the treatment outcomes from 44.4% of mortality in years 2002–2003 to less than 15.0% of mortality after year 2006 [13–15]. Once these criteria were met, patients were enrolled in this study, and no further inclusion or exclusion criteria were used. The aggregate number of enrolled patients in this 4-year study was 39.

### Measurements

We summarized the collected data at the time of study enrollment, which included patient age, gender, history, predisposing factors, presenting signs and symptoms, location of infection, laboratory findings, bacteriologic results, length of stay and outcomes such as survival and limb salvage. Regarding the laboratory findings, we collected the data at the time of patient arrival at emergency department (admission data) and that data, checked during hospitalization, having greatest difference with normal reference values (extreme data). Admission data were used to calculate the LRINEC (laboratory risk indicator for necrotizing fasciitis), MELD (model for end-stage liver diseases) and MELD-Na (modified MELD including sodium) scores on admission. Using both the admission and the extreme data, we further synthesized the ΔLRINEC, ΔMELD and ΔMELD-Na scores (i.e., differences between the admission and the extreme scores) for possibly predicting case-fatality. Case-fatality in this study was defined as death during hospitalization.

### Statistical analysis

Descriptive data were presented as means with standard errors for continuous variables and numbers with percentages for categorical variables. Wilcoxon rank sum test was used to compare continuous variables, and Fisher exact test was used to compare dichotomous variables. The diagnostic performance of the index scoring models to discriminate between deceased patients from surviving patients was evaluated using Receiver Operating Characteristic (ROC) curve analysis. Accuracy was measured by the area under the ROC curve (AUC). The optimal cutoff value for predicting case-fatality was identified as the score giving the best Youden index [maximum (sensitivity + specificity -1)] for the scoring model [16]. The sensitivity, specificity, positive predictive value (PPV), and negative predictive value (NPV) were calculated at the optimal cutoff value for the scoring system assessment. Additionally, odds ratios (ORs) and 95% confidence intervals (CIs) were also calculated in the logistic regression model. A two-tailed *p* value of < 0.05 was considered significant. All statistic analyses were conducted using the Statistical Package for the Social Sciences (SPSS, version 17.0. SPSS Inc., Chicago, IL, USA) and/or the MedCalc Statistical Software (version 9.5, Broekstraat, Mariakerke, Belgium).

### Ethics statement

The datas were analyzed after approval by the ethic committee (Institutional Review Board) of the Chang Gung Memorial Hospital in Taiwan. We did not obtain informed consent from the patient due to a statement of this committee, that analyzing patient data retrospectively requires no informed consent.

## Results

### Patient characteristics and related variables (Table 1)

**Table 1:** Laboratory findings on admission and the extreme data during hospitalization in 39 *V*. *vulnificus*-infected patients treated between 2007 and 2010.

Variables	Admission Data	Extreme Data	*P* Value^†^
	(n = 39)	(n = 39)	
**CRP** ^**a**^ **(mg/dL)**	77.5 ± 12.7	157.0 ± 16.2	< 0.001*
**WBC** ^**b**^ **(× 103 per mm^3^)**	12.8 ± 0.9	18.7 ± 1.0	< 0.001*
**Band (%)**	7.7 ± 1.4	11.5 ± 1.4	< 0.001*
**Hemoglobin (g/dL)**	13.5 ± 0.3	10.2 ± 0.3	< 0.001*
**Glucose (mg/dL)**	149.6 ± 10.7	152.6 ± 10.6	0.109
**Sodium (meq/L)**	135.5 ± 0.6	132.3 ± 0.8	< 0.001*
**Creatinine (mg/dL)**	1.78 ± 0.16	2.04 ± 0.19	0.001*
**Bilirubin (mg/dL)**	1.93 ± 0.41	2.38 ± 0.54	0.012*
**INR** ^**C**^	1.29 ± 0.06	1.41 ± 0.09	0.002*
**Albumin (g/dL)**	2.35 ± 0.07	2.09 ± 0.07	< 0.001*
**LRINEC** ^**d**^ **score**	3.4 ± 0.4	7.3 ± 0.5	< 0.001*
**MELD** ^**e**^ **score**	15.1 ± 1.1	17.1 ± 1.4	< 0.001*
**MELD-Na** ^**f**^ **score**	17.7 ± 1.1	20.9 ± 1.3	< 0.001*

Data are presented as mean ± SE, unless otherwise indicated.

^†^ Wilcoxon rank sum test, unless otherwise indicated.

* The difference is significant (p < 0.05).

^a^ CRP, C-reactive protein.

^b^ WBC, white blood cells.

^c^ INR, international normalized ratio.

^d^ LRINEC, laboratory risk indicator for necrotizing fasciitis.

^e^ MELD, model for end-stage liver disease.

^f^ MELD-Na, modified model for end-stage liver disease including sodium.

Thirty-nine patients with VNSSTIs were enrolled in this 4-year-long study. All patients had undergone the above-mentioned treatment protocol [14]. The mean age of our patients was 65.7 ± 11.3 years old; thirty (76.9%) patients were male, and nine (23.1%) were female. Among the involved lesion limbs, lower limbs were predominant (56.4%). Eight (20.5%) episodes, including one fatal case, were categorized as primary sepsis with secondary skin manifestations; whereas thirty-one (79.5%) episodes, including 4 fatal cases, were manifested as necrotizing wound infection with/without bacteremia. On arrival at emergency department, fourteen (35.9%) patients had a fever (a temperature of > 38.5°C or 101.3°F) and twenty-three (59.0%) patients were hypotensive with a systolic blood pressure of ≤ 90 mmHg. Thirty-one (79.5%) patients needed medical care in an intensive care unit (ICU). The mean hospitalization day in this study was 38.5 ± 3.1 days. Most (92.3%) patients were immunocompromised and liver dysfunction was the leading (64.1%) underlying disease rendering these patients immunocompromised.

Regarding the admission data, our patients with VNSSTIs had high CRP (77.5 ± 12.7 mg/dL), leukocytosis with left shift (12.8 ± 0.9 × 10^3^ per mm^3^ with 7.7 ± 1.4% of band form), hyperglycemia (149.6 ± 10.7 mg/dL), hypoalbuminemia (2.35 ± 0.07 g/dL) and increased levels of serum creatinine (1.78 ± 0.16 mg/dL), the INR (1.29 ± 0.06), and serum bilirubin (1.93 ± 0.41 mg/dL). The calculated LRINEC, MELD, and MELD-Na scores on admission were, therefore, 3.4 ± 0.4, 15.1 ± 1.1, and 17.7 ± 1.1 points, respectively. Paying particular attention to the extreme data, we observed that our patients would have temporary or progressive deterioration of nearly all their scores and lab values after admission except for their sugar level (*p* = 0.109). Besides, there was a significant trend for these patients to develop anemia (10.2 ± 0.3 g/dL, *p* < 0.001), hyponatremia (132.3 ± 0.8 meq/L, *p* < 0.001) and hypoalbuminemia (2.09 ± 0.07 g/dL, *p* < 0.001) during hospitalization. The highest LRINEC, MELD and MELD-Na scores during hospitalization was calculated to be 7.3 ± 0.5, 17.1 ± 1.4, and 20.9 ± 1.3 points (all *p* < 0.001), respectively.

### Group comparison of laboratory findings (Tables 2 and 3)

**Table 2:** Group comparison of laboratory findings on admission and the extreme data during hospitalization.

	Admission Data		
	Surviving Patients	Deceased Patients	
Variables	(n = 34)	(n = 5)	*P* Value^†^
**CRP** ^**a**^ **(mg/dL)**	79.7 ± 14.3	62.4 ± 23.6	0.769
**WBC** ^**b**^ **(× 103 per mm^3^)**	13.7 ± 1.0	7.4 ± 2.2	0.042*
**Band (%)**	5.9 ± 1.0	19.7 ± 6.8	0.045*
**Hemoglobin (g/dL)**	13.4 ± 0.3	13.7 ± 0.9	0.769
**Glucose (mg/dL)**	154.6 ± 11.9	116.2 ± 14.4	0.141
**Sodium (meq/L)**	136.0 ± 0.6	132.3 ± 2.0	0.048*
**Creatinine (mg/dL)**	1.58 ± 0.13	3.12 ± 0.67	0.013*
**Bilirubin (mg/dL)**	1.32 ± 0.14	6.08 ± 2.56	0.001*
**INR** ^**c**^	1.22 ± 0.04	1.76 ± 0.33	0.037*
**Albumin (g/dL)**	2.39 ± 0.07	2.02 ± 0.21	0.074

Data are presented as mean ± SE, unless otherwise indicated.

^†^ Wilcoxon rank sum test, unless otherwise indicated.

* The difference is significant (p < 0.05).

^a^ CRP, C-reactive protein.

^b^ WBC, white blood cells.

^c^ INR, international normalized ratio.

**Table 3:** Group comparison of laboratory findings on admission and the extreme data during hospitalization.

	Extreme Data		
	Surviving Patients	Deceased Patients	
Variables	(n = 34)	(n = 5)	P Value^†^
**CRP** ^**a**^ **(mg/dL)**	160.8 ± 18.3	131.3 ± 20.6	0.450
**WBC** ^**b**^ **(× 103 per mm^3^)**	18.0 ± 1.1	23.5 ± 2.6	0.074
**Band (%)**	9.6 ± 1.1	24.3 ± 5.8	0.007*
**Hemoglobin (g/dL)**	10.6 ± 0.4	8.0 ± 0.6	0.014*
**Glucose (mg/dL)**	156.3 ± 11.9	127.2 ± 13.5	0.424
**Sodium (meq/L)**	132.7 ± 0.9	129.2 ± 2.0	0.085
**Creatinine (mg/dL)**	1.77 ± 0.15	3.89 ± 0.53	0.002*
**Bilirubin (mg/dL)**	1.43 ± 0.18	8.82 ± 2.79	0.001*
**INR** ^**c**^	1.32 ± 0.09	2.03 ± 0.31	0.026*
**Albumin (g/dL)**	2.16 ± 0.07	1.62 ± 0.21	0.019*

Data are presented as mean ± SE, unless otherwise indicated.

^†^ Wilcoxon rank sum test, unless otherwise indicated.

* The difference is significant (p < 0.05).

^a^ CRP, C-reactive protein.

^b^ WBC, white blood cells.

^c^ INR, international normalized ratio.

In this case series, thirty-four (87.2%) patients survived (group 1: surviving patients) whereas five (12.8%) patients died (group 2: deceased patients). The mean age of surviving patients was 66.4 ± 11.0 years old and that of deceased patients was 61.6 ± 14.3 years old (*p* = 0.366). Comparison of these two groups revealed no significant differences not only in CRP, hemoglobin, glucose and albumin levels on admission (all *p* ≥ 0.074) but also in the extreme data of CRP, WBC, glucose and sodium levels during hospitalization (all *p* ≥ 0.074). On admission, the deceased patients had higher rates of leukopenia (*p* = 0.042), left shift (*p* = 0.045), and hyponatremia (*p* = 0.048) and higher levels of serum creatinine (*p* = 0.013), the INR (*p* = 0.037) and serum bilirubin (*p* = 0.001) than the surviving patients. Paying particular attention to the extremes of change in lab values during hospitalization, we further found that the deceased patients would develop more severe forms of anemia (*p* = 0.014) and hypoalbuminemia (*p* = 0.019) than the surviving patients.

### Group comparison of LRINEC, MELD, and MELD-Na scores (Table 4)

**Table 4:** Group comparison of LRINEC, MELD and MELD-Na scores on admission, the extreme scores during hospitalization, and their differences.

	Surviving Patients	Deceased Patients	
Variables	(n = 34)	(n = 5)	*P* Value^†^
**Admission Score**			
** LRINEC** ^**a**^ **score**	3.4 ± 0.4	4.0 ± 0.8	0.229
** MELD** ^**b**^ **score**	13.3 ± 0.8	27.2 ± 4.9	0.002*
** MELD-Na** ^**c**^ **score**	16.0 ± 0.8	28.8 ± 4.4	0.008*
**Extreme Score**			
** LRINEC** ^**a**^ **score**	7.0 ± 0.6	9.0 ± 0.8	0.213
** MELD score**	14.7 ± 1.1	32.8 ± 3.3	0.001*
** MELD-Na score**	18.9 ± 1.1	34.4 ± 2.5	0.001*
**Difference**			
** Δ** ^**d**^ **LRINEC score**	3.7 ± 0.5	5.0 ± 0.9	0.253
** Δ MELD score**	1.5 ± 0.7	5.6 ± 1.6	0.002*
** Δ MELD-Na score**	2.9 ± 0.6	5.6 ± 2.1	0.140

Data are presented as mean ± SE, unless otherwise indicated.

^†^ Wilcoxon rank sum test, unless otherwise indicated.

* The difference is significant (p < 0.05).

^a^ LRINEC, laboratory risk indicator for necrotizing fasciitis.

^b^ MELD, model for end-stage liver disease.

^c^ MELD-Na, modified model for end-stage liver disease including sodium.

^d^ Δ, difference between the admission and the extreme scores.

Comparison of the two groups revealed no differences in the LRINEC scores, including the initial scores synthesized on admission and the extreme scores during hospitalization (*p* ≥ 0.213). Not only on admission but also during hospitalization, the deceased patients had higher MELD (both *p* ≤ 0.002) and MELD-Na (both *p* ≤ 0.008) scores than the surviving patients. Regarding the differences between the admission and the extreme scores, we found that there was a significant difference in the ΔMELD scores (*p* = 0.002), but neither in the ΔLRINEC scores nor in the ΔMELD-Na scores (*p* ≥ 0.140).

### Predicting mortality risk with ROC curve analysis (Tables 5 and 6)

**Table 5:** Area under the receiver operating characteristic (ROC) curve for predicting mortality risk in 39 *V*. *vulnificus*-infected patients treated between 2007 and 2010.

Variables	AUC^e^	95% CI^f^	*P* Value
**LRINEC** ^**a**^ **score**	0.665	0.403–0.926	0.240
**MELD** ^**b**^ **score**	0.929	0.818–1.000	0.002*
**MELD-Na** ^**c**^ **score**	0.871	0.662–1.000	0.008*
**Δ** ^**d**^ **MELD score**	0.897	0.772–1.000	0.005*
**Δ MELD-Na score**	0.703	0.463–0.942	0.147

* P value < 0.05 is significant and all analysis was done by logistic regression model in SPSS 17.0.

^a^ LRINEC, laboratory risk indicator for necrotizing fasciitis.

^b^ MELD, model for end-stage liver disease.

^c^ MELD-Na, modified model for end-stage liver disease including sodium.

^d^ Δ, difference between the admission and the extreme scores.

^e^ AUC, area under the receiver operating characteristic curve.

^f^ CI, confidence interval.

**Table 6:** Odds ratio of the index variables in relation to the case fatality.

Variables	OR^e^	95% CI^f^	*P* Value
**LRINEC** ^**a**^ **score ≥ 6**	1.45	0.13–15.79	1.000
**MELD** ^**b**^ **score ≥ 15**	1.31	1.03–1.67	0.082
**MELD score ≥ 16**	1.42	1.04–1.93	0.025*
**MELD score ≥ 17**	13.00	1.27–133.64	0.042*
**MELD score ≥ 20**	64.00	4.68–875.43	< 0.001*
**MELD-Na** ^**c**^ **score ≥ 15**	1.24	1.03–1.49	0.236
**MELD-Na score ≥ 19**	3.60	0.52–24.93	0.397
**MELD-Na score ≥ 20**	18.67	1.76–198.10	0.015*
**Δ** ^**d**^ **LRINEC score ≥ 3**	2.48	0.25–24.65	0.768
**Δ LRINEC score ≥ 5**	2.42	0.36–16.50	0.662
**Δ MELD score ≥ 1**	1.36	1.04–1.78	0.048*
**Δ MELD score ≥ 2**	13.00	1.27–133.64	0.042*
**Δ MELD-Na score ≥ 3**	1.50	0.22–10.14	1.000
**Δ MELD-Na score ≥ 5**	3.11	0.42–22.87	0.574

* P value < 0.05 is significant and all analysis was done by logistic regression model in SPSS 17.0.

^a^ LRINEC, laboratory risk indicator for necrotizing fasciitis.

^b^ MELD, model for end-stage liver disease.

^c^ MELD-Na, modified model for end-stage liver disease including sodium.

^d^ Δ, difference between the admission and the extreme scores.

^e^ OR, odds ratio;

^f^ CI, confidence interval.

**Fig 1:**
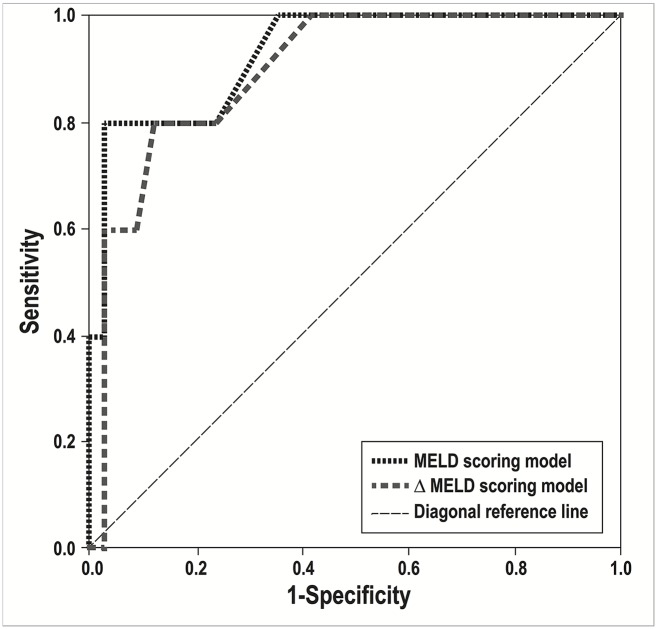
Receiver operating characteristic (ROC) curve for predicting case fatality risk in 39 *V*. *vulnificus*-infected patients. The areas under the ROC curve (AOC) of the MELD and ΔMELD are 0.929 (95% CI = 0.818–1.000; *p* = 0.002) and 0.897 (95% CI = 0.772–1.000; *p* = 0.005), respectively. The diagonal reference line indicates no discrimination.

The AUC estimates for the MELD, ΔMELD and MELD-Na scoring models were 0.929 (95% CI: 0.818–1.000, *p* = 0.002), 0.897 (95% CI: 0.772–1.000, *p* = 0.005) and 0.871 (95% CI: 0.662–1.000, *p* = 0.008), respectively (Fig 1). The above-mentioned scoring models were, therefore, considered to be good-to-excellent at discriminating deceased patients from surviving patients in the current study. In contrast, the discriminative power of the LRINEC and ΔMELD-Na scoring models was considered to be poor. Using Youden index, the optimal cutoff values of the MELD and ΔMELD scoring models for predicting case-fatality risk were 20 and 2, respectively. A significantly increased case-fatality rate (66.7%) was observed for MELD score ≥ 20 on admission, whereas for total patients in this study, the case-fatality rate was 12.8%. At a MELD cutoff value ≥ 20, the model had a sensitivity of 80%, a specificity of 97%, PPV of 80%, and NPV of 97%. At a ΔMELD cutoff value of ≥ 2, the model had a sensitivity of 80%, a specificity of 88%, PPV of 50%, and NPV of 97%. Patients with MELD score ≥ 20 on admission had a significantly higher case-fatality risk compared to those with MELD score < 20 (OR = 64, 95% CI: 4.68–875.43, *p* < 0.001). Besides, patients with ΔMELD score ≥ 2 during hospitalization had a significantly higher case-fatality risk compared to those with ΔMELD score < 2 (OR = 13, 95% CI: 1.27–133.64, *p* = 0.042).

## Discussion

Liver dysfunction can be associated with infections and complications from *V*. *vulnificus*. It is implicated in the host defense, participating in the clearance of the infectious agents and/or their products. Sepsis also induces liver damage through hemodynamic alterations or through direct or indirect assault on the hepatocytes or through both [6]. Accordingly, liver dysfunction is considered as one of the main components that contribute the severity and mortality of VNSSTIs. However, there is a lack of simple and reliable evaluation tools allowing detection of progressing liver dysfunction during medical care of patients with VNSSTIs, thus improving the treatment outcome in this group. To the best of our knowledge, this study is the first to report on the performance of MELD scoring system and its variants in *V*. *vulnificus*-infected patients. We have learned that the MELD scoring model is an effective risk stratification indicator at the time of admission and the ΔMELD scoring model is an excellent condition monitor during hospitalization. The AUC for these two models approximated 0.9 by using the ROC curve analysis, demonstrating that they had a good discriminatory ability for predicting case-fatality in patients with VNSSTIs. Considering the rapidly progressing nature and the highly lethal consequences of the infection, the MELD/ΔMELD scoring model, having a good sensitivity and specificity (all > 80%), can help clinicians timely and confidently stratify and monitor these patients during the whole clinical course.

In the present study of 39 patients with VNSSTIs, we identified optimal cutoff points of ≥ 20 and ≥ 2 in the MELD and ΔMELD prediction models, respectively. Patients with MELD score ≥ 20 had a high case-fatality rate of 66.7% and were associated with a 64-fold increased odds for death during hospitalization. After admission for medical care, those with ΔMELD score ≥ 2 were associated with a 13-fold increased odds for case-fatality. These results may be valuable and provide the basis of clinical practice guidelines and recommendations for monitoring patients with VNSSTIs. Some researchers reported that the MELD-Na/ΔMELD-Na scoring model may have a better fit for mortality prediction compared to the MELD/ΔMELD scoring model alone [17]; however, we did not observe a superiority of the MELD-Na/ΔMELD-Na model in predicting the case-fatality of patients with VNSSTIs. Wong et al. advocated using the LRINEC scoring model to clinically detect early cases of necrotizing fasciitis [18, 19]. They reported that LRINEC scores of ≥ 8, ≥ 6 and ≤ 5 indicate high, intermediate and low, respectively, risk of necrotizing skin and soft tissue infections. The current data revealed that the LRINEC scoring model may not be applicable for the medical care of patients with VNSSTIs not only due to the underestimation of the infection severity (3.4 ± 0.4 and 4.0 ± 0.8 for the surviving and the deceased patients, respectively) but also because of its poor discriminatory power in identifying which septic patients have increased mortality risk (AUC = 0.665 [95% CI: 0.403–0.926; *p* = 0.240]).

It is interesting that there was a significant trend for total patients with VNSSTIs to develop anemia (10.2 ± 0.3 g/dL, *p* < 0.001), hyponatremia (132.3 ± 0.8 meq/L, *p* < 0.001) and hypoalbuminemia (2.09 ± 0.07 g/dL, *p* < 0.001) during hospitalization. Moreover, the deceased patients would suffer from more severe forms of anemia (*p* = 0.014), of hypoalbuminemia (*p* = 0.019), but not of hyponatremia (*p* = 0.085) than the surviving patients under the current treatment protocol [14]. Possible explanations are the preexisting protein calorie malnutrition and the infection/inflammation induced consequences such as increased capillary leak and inhibition of erythropoiesis [20]. In critically ill patients, the body’s erythropoietic response to anemia is blunted as a consequence of diminished iron availability and the direct inhibitory effects of inflammatory cytokines [21]. Low serum albumin levels predispose these patients to have reduced potential for oxygen radical scavenging, which in turn aggravates the sepsis and then leads back to hypoalbuminemia in a more severe form via decreased albumin synthesis and altered distribution [22, 23]. Corona et al. [24] provided evidence from a meta-analysis indicating that moderate-to-severe hyponatremia is significantly associated with an increased risk of overall mortality. Although not statistically significant, the deceased patients in the current study had advanced to moderate hyponatremia even under our aggressive treatment. It is uncertain that whether correction of hemoglobin, albumin and sodium levels through parenteral supplement would really help to reduce mortality; however, further studies about the effect of exogenous erythropoietin, albumin, and vasopressin should be considered in these critically ill patients with VNSSTIs [25–29].

It is not surprising that our patients had temporary or progressive deteriorations of the lab values of serum creatinine, the INR and serum bilirubin (all *p* ≤ 0.012) during hospitalization, which combinedly constitute the three components of the MELD scoring model. The MELD score formula is: 3.8 [log_e_ serum bilirubin (mg/dL)] + 11.2 [log_e_ INR] + 9.6 [log_e_ serum creatinine 9mg/dL)] + 6.4 [9]. Serum bilirubin concentration is a well established marker of the hepatic synthetic function, although it represents excretory function. Prothrombin time and the INR reflect coagulopathy associated with synthetic dysfunction in patients with advanced liver dysfunction. Because renal dysfunction in patients with liver disease is an ominous sign for mortality, serum creatinine concentration is heavily weighted in the MELD score equation. In interpreting the MELD score in hospitalized patients, Wiesner et al. [30] had reported that the 3-month mortality was more than 19.6% in patients with MELD score ≥ 20. In contrast, the current study on patients with VNSSTIs revealed that the case-fatality rate was as high as 66.7% for patients with MELD score ≥ 20 on admission, whereas for total patients the case-fatality rate was only 12.8%. Botta et al. [9] had used the MEGX test, a tool for the real-time assessment of hepatic function, to confirm the fact that an increase in MELD score is associated with a decrease in residual liver function. In critically ill patients after polytrauma or sepsis, several studies had further shown that a decrease in MEGX test values (i.e., hepatic functional impairment) is associated with an enhanced systemic inflammatory response and an enhanced risk for the development of MODS and a poor outcome [31]. We, therefore, recommend that an earlier and better identification of *V*. *vulnificus*-infected patients with liver dysfunction is warranted and may be the way to evaluate new therapeutic strategies and further improve the prognosis of sepsis.

Despite the promising preliminary results of applying the MELD/ΔMELD scoring model to medical care of patients with VNSSTIs, this investigation has limitations. First, this is a retrospective study harboring all the potential drawbacks implicit in such a study design. Second, the number of patients included during the study period was relatively small and the study may have lacked power to detect the statistical differences in all prognostic factors among subsets of patients. The uncommon occurrence and fulminant nature of VNSSTIs in humans makes large-scale prospective studies quite difficult to conduct in a measurable period. Finally, this investigation is lacking in physiological parameters such as those contained in the acute physiology and chronic health evaluation (APACHE) II scoring system [32]. However, the APACHE II score is a relatively complex and time-consuming tool that requires more physiological and laboratory parameters, whereas the variables of the MELD scoring model are relatively easily available at the time of admission and during hospitalization. Besides, the present study was designed to focus and concentrate on the performance of the MELD scoring model and its variants in *V*. *vulnificus*-infected patients, leaving other considerations aside.

In conclusion, we recommend that the MELD/ΔMELD scoring model is an effective risk stratification indicator at the time of admission and also an excellent condition monitor during hospitalization for medical care of acutely and/or critically ill patients with VNSSTIs. In addition to careful history taking and physical examination, this scoring model could help clinicians identify patients who might have progressing liver dysfunction and should receive proper disposition or undergo specific therapeutic interventions. Besides, temporary or progressive anemia, hypoalbuminemia and/or hyponatremia would occur even with the current aggressive treatment. Further studies about the effect of exogenous erythropoietin, albumin, and vasopressin should be considered in these critically ill patients with VNSSTIs.
